# Tactile-Foot Stimulation Can Assist the Navigation of People with Visual Impairment

**DOI:** 10.1155/2015/798748

**Published:** 2015-03-22

**Authors:** Ramiro Velázquez, Edwige Pissaloux, Aimé Lay-Ekuakille

**Affiliations:** ^1^Mecatrónica y Control de Sistemas (MCS), Universidad Panamericana, 20290 Aguascalientes, MEX, Mexico; ^2^Institut des Systèmes Intelligents et de Robotique (ISIR), Université Paris 6, 75005 Paris, France; ^3^Dipartimento d'Ingegneria dell'Innovazione (DII), Università del Salento, 73100 Lecce, Italy

## Abstract

*Background.* Tactile interfaces that stimulate the plantar surface with vibrations could represent a step forward toward the development of wearable, inconspicuous, unobtrusive, and inexpensive assistive devices for people with visual impairments.* Objective.* To study how people understand information through their feet and to maximize the capabilities of tactile-foot perception for assisting human navigation.* Methods.* Based on the physiology of the plantar surface, three prototypes of electronic tactile interfaces for the foot have been developed. With important technological improvements between them, all three prototypes essentially consist of a set of vibrating actuators embedded in a foam shoe-insole. Perceptual experiments involving direction recognition and real-time navigation in space were conducted with a total of 60 voluntary subjects.* Results.* The developed prototypes demonstrated that they are capable of transmitting tactile information that is easy and fast to understand. Average direction recognition rates were 76%, 88.3%, and 94.2% for subjects wearing the first, second, and third prototype, respectively. Exhibiting significant advances in tactile-foot stimulation, the third prototype was evaluated in navigation tasks. Results show that subjects were capable of following directional instructions useful for navigating spaces.* Conclusion.* Footwear providing tactile stimulation can be considered for assisting the navigation of people with visual impairments.

## 1. Introduction

Designing navigational assistive interfaces for people with visual impairment involves two major challenges. (1) Information must be presented in a nonvisual form and in a simple, intuitive, and fast to understand manner. (2) The interface must be lightweight and inconspicuous and ensure an autonomy of at least a regular walking journey.

Many navigational assistive interfaces for the visually impaired can be found in the literature. Many of them use an acoustic information presentation method [1–3]. However, a major drawback of such systems is that acoustic feedback causes distraction and interferes with the user's normal hearing activities. Some other interfaces use tactile feedback with the aim of avoiding user distraction with musical tones [4–6]. A major drawback of touch stimulation systems is that information displayed can be complex and slow to understand. Readers are referred to [7, 8] for two comprehensive and reasonably updated surveys on electronic interfaces for the visually impaired.

Most of the interfaces that exploit tactile stimulation are destined to the fingertips and palms. They are usually used in conjunction with the primary aids (white cane and guide dog). Unfortunately, as they require constant hand interaction, they quickly cause bodily fatigue and user rejection.

During the last years, we have been exploring the feasibility of providing tactile feedback via the feet. We have developed three prototypes of tactile interfaces for the foot that allow hands-free interaction and that are worn rather than carried by the user. With important improvements between them, all three prototypes essentially consist of a set of actuators embedded in a foam shoe-insole that provide vibrotactile stimulation to the plantar surface. These inexpensive devices address the two major challenges of assistive interfaces aforementioned: (1) they present vibrating patterns that are simple and fast to understand and (2) they can be further inserted into a shoe, thus becoming inconspicuous, visually unnoticeable assistive devices.

Experimental perceptual studies have been already conducted in the past with the first prototype in sighted and visually impaired users [9]. Our results indicate that people actually understand information displayed to the plantar surface of the foot. This paper briefly recalls those results and reports our progresses in tactile-foot stimulation with two new improved devices. In particular, prototypes are evaluated and compared in direction rendering which is the basis for real-world navigation.

The rest of the paper is organized as follows: Section 2 presents background information on tactile-foot stimulation.  Section 3 introduces the first prototype developed and its evaluation in direction rendering.  Section 4 presents a technologically improved second prototype and evaluates the pertinence of these improvements in the same task.  Section 5 presents the third prototype and its evaluation and compares the results with those obtained with the two previous prototypes. Real-time navigation of test participants is shown to demonstrate the effectiveness of the third prototype. Finally, Section 6 concludes summarizing the paper's main contributions as well as future work perspectives.

## 2. Background

Tactile paving is the most representative example of a tactile-foot stimulation system. It consists of regularly textured ground surface indicators in the form of patterns of raised domes or bars. Its purpose is to assist visually impaired pedestrians with their navigation (bar pattern) and alert them of hazards or obstacles in the immediate location (dome pattern).

Literature addressing electronic interfaces that display tactile information to the feet can be found in several domains.

In man-machine interaction, tactile-foot stimulation can be found in certain user control interfaces: car pedals [10], fitness gear [11], dental equipment [12], music instruments [13], and so forth. They provide vibrating cues to alert users of well-defined machine situations.

In human-computer interaction, tactile-foot stimulation is mostly oriented towards recreating virtual environments. Papetti et al. proposed a combined audio-tactile system that provides the sensation of walking over different grounds [14]. Okamoto et al. developed a footstep display that recreates the sensation of stepping on fragile structures, such as paper, aluminum, and polypropylene [15]. Jayakumar et al. introduced a haptic foot-based interface providing the sensation of mechanical impacts during virtual walking [16]. Nordahl et al. explored the use of tactile-foot stimulation, together with visual and auditory stimuli, to generate vertical illusory self-motion during exposure to a virtual environment depicting an elevator [17].

In biomedical engineering, tactile-foot stimulation has been mostly explored in the control of human posture and standing balance [18, 19] as well as locomotion [20]. To our knowledge, it has not been yet explored in assistive devices.

## 3. First Prototype 

### 3.1. Device

**Figure 1:**
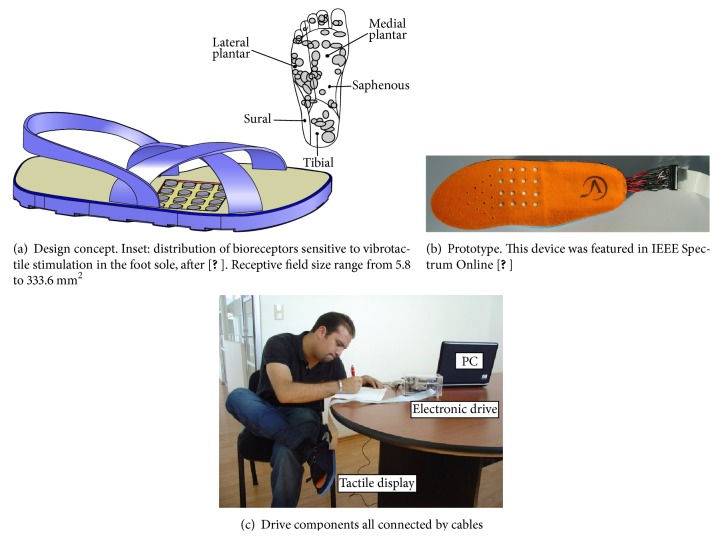
First prototype of tactile interface for the foot.

Based on the physiology of the plantar surface of the foot, which indicates that the great majority of the bioreceptors sensitive to vibrotactile stimulation are located on the medial and lateral areas [21], a first tactile interface consisting of a 16-point array of actuators was conceived (Figure 1(a)).

The prototype integrated all 16 actuators in a commercial inexpensive foam shoe-insole with 10 mm pin spacing (Figure 1(b)). The pins' contact surface with the skin was 7 mm^2^ and targeted the bioreceptors of smallest receptive field size (Figure 1(a) inset). Actuators provided axial forces up to 13 mN and vibrating frequencies between 10 and 55 Hz. Each vibrator was independently controlled with a specific vibrating frequency command. This first prototype was meant to be used on the right foot and it was controlled by a computer through an electronic unit. All subsystems were connected by cables [9] (Figure 1(c)).

### 3.2. Evaluation 

#### 3.2.1. Study Participants and Experimental Procedure

Twenty undergraduate students (10 men and 10 women) at Panamericana University participated voluntarily in the experiment. All gave their consent in agreement with the university ethics guidelines. No special criteria were used to select them but availability. All participants were healthy sighted with no known impairments in tactile sensory or cognitive functions. Their ages ranged from 18 to 24 years with an average age of 20.5.

During the experiment, the subjects were seated wearing the tactile interface on the right foot (Figure 1(c)). For hygiene, all subjects were requested to use socks. Before the experiment, they were totally naive about all aspects of the test and were given general instructions concerning the task. A short familiarization time was granted prior to the tests. During this time, the subjects tested different vibration frequencies and had the opportunity to choose a preferred one. All 20 subjects chose 55 Hz, the maximum vibration frequency of the actuators.

#### 3.2.2. Direction Recognition Experiment

The purpose of this test was to determine whether the subjects could recognize directions with the first prototype.

**Figure 2:**
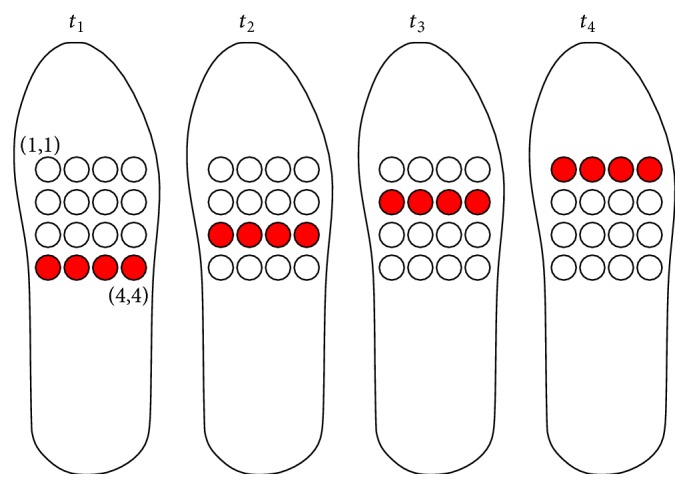
Prototype 1: schedule of activation of the vibrating actuators for the direction recognition task. Example for North.

*Method.* A dynamic straight line was presented to the 20 subjects. Four patterns were chosen: North N (a line moving from the last row to the first one), South S (the inverse), West W (a line moving from the last column to the first one), and East E (the inverse) (Figure 2). Note that these tactile inputs refer to relative directions where North is always associated with the direction the subject is heading. They do not necessarily imply actual cardinal directions.

A set of 14 directions was presented to the subjects in one trial: S-N-E-W-S-E-N-W-E-S-W-N-S-E. This set takes into account all possible transitions between directions. Subjects were asked to report the direction perceived with no time restriction. Upon request, they could have the vibrating pattern refreshed on the interface. 

*Results.* Subjects required from 8 to 10 min of training to get used to the patterns. Results obtained are presented in confusion matrix in Table 1. The average recognition rates were 71.92%, 71.05%, 80.24%, and 80.7% for N, S, W, and E, respectively. The average accuracy of confusion matrix (Table 1) is 76%. Note an overall good performance.

During the test, 12 of the 20 subjects requested to have at least one direction of the set refreshed. We noticed that users felt correctly the vibrations but sometimes they were unable to locate them precisely on their foot-sole.

## 4. Second Prototype 

### 4.1. Device

**Figure 3:**
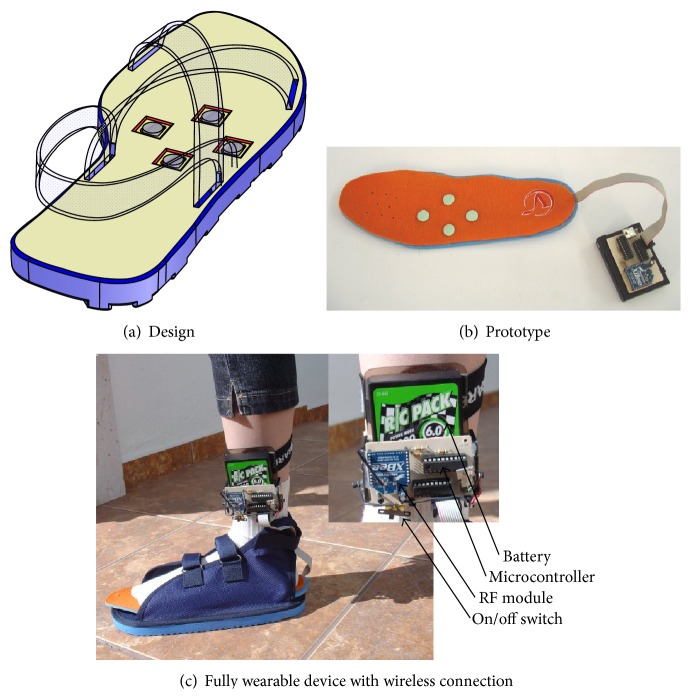
Second prototype of tactile interface for the foot.

With the aim of improving user perception, a second prototype was envisaged (Figure 3(a)). Note that this design reduced the number of vibrators from 16 to 4 and that was meant to stimulate the same plantar areas: medial and lateral.

This design modification addressed the observations made while testing with prototype 1: people do understand information displayed on the plantar surface of the foot. However, the foot is not capable of precise discrimination; that is, people cannot accurately distinguish which actuator is actually vibrating. From a technological point of view, it is not worthy then to integrate a large number of actuators in the interface. Work must be done at tactile rendering level.

Figure 3(b) shows the second prototype of tactile interface for the foot. Vibrators were arranged in a diamond-like shape with 35 mm side-length. Their characteristics are identical to those used in the previous prototype (13 mN, 10–55 Hz). However, the pins' contact surface with the foot-sole was increased to 133 mm^2^ to enclose more small receptive field size bioreceptors and stimulate them together with medium receptive field size bioreceptors. This design was meant to be used on the left foot.

This device is completely wearable.  Figure 3(c) illustrates how it is worn by a user. Note that battery, RF (radio-frequency) transmission module, and control circuitry are all embedded in an electronic module that the user carries comfortably attached to the ankle. Experimental tests revealed a 6 h continuous operation of the device and a 100 m communication distance range with a computer.

### 4.2. Evaluation

#### 4.2.1. Study Participants and Experimental Procedure

A group of 20 undergraduate students (14 men and 6 women) were invited to participate in the experiment. None of them had participated previously evaluating prototype 1. All subjects were healthy sighted with no known impairments in tactile sensory or cognitive functions. Their ages ranged from 19 to 23 years with an average age of 20.2. All gave their consent in agreement with the university ethics guidelines.

The same procedure was followed: subjects were seated wearing socks with the interface now on the left foot. There was no previous knowledge of any of the aspects of the experiment. A short familiarization time with the interface was granted. Vibrating actuators were working at 55 Hz.

#### 4.2.2. Direction Recognition Experiment

The purpose of this test was to determine whether the subjects could obtain better recognition rates upon the use of the second prototype and new vibrating patterns.

*Method.* Each one of the four contact pins of the interface was set to represent a cardinal point (again, not necessarily representing the actual one). A direction is encoded in five sequences (*t*_1_–*t*_5_) as follows: three consecutive short vibrations in the corresponding contact pin, then a short vibration in the opposite contact pin, and again a short vibration in the correct contact pin.

**Figure 4:**
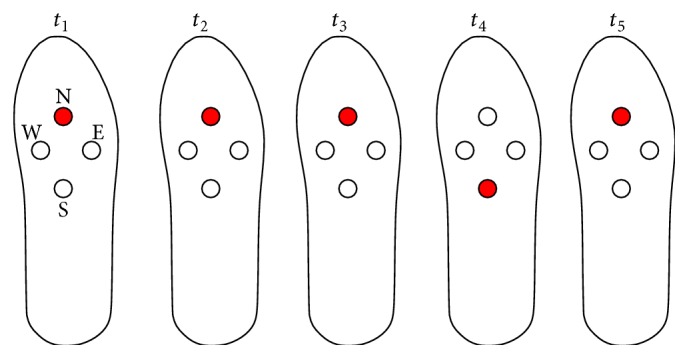
Prototypes 2 and 3: schedule of activation of the vibrating actuators for the direction recognition task. Example for North.

Figure 4 shows, for example, the codification for North. Note that the contact pin N vibrates three times, then S once, and again N. The same set of 14 directions was presented to the subjects in one trial. All 20 subjects were asked to report the direction perceived with no time restriction. Upon request, they could have the direction pattern refreshed on the interface.

*Results.* Subjects required from 3 to 6 min of training to get used to the patterns. Results obtained are presented in confusion matrix in Table 2. The average recognition rates were 91.65%, 91.25%, 78.75%, and 91.65% for N, S, W, and E, respectively. The average accuracy of confusion matrix (Table 2) is 88.3%. Note that recognition rates improved for N, S, and E while for W, it was practically the same as in prototype 1.

Note that tactile patterns used in the second prototype are easier to recognize. Now, the opposite direction is also displayed to indicate a direction. This provides a reliable reference to identify points of vibration. As it can be seen from the recognition rates, the fact of displaying both correct and opposite directions in the same tactile pattern eases recognition and does not confuse the user.

## 5. Third Prototype 

### 5.1. Device

**Figure 5:**
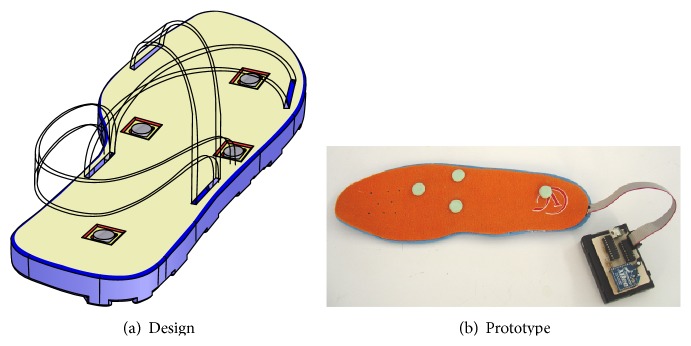
Third prototype of tactile interface for the foot.

Figure 5(a) shows the conceptual representation of the third prototype. Note that the only modification is that contact pin S stimulates now the tibial plantar area (Figure 1(a) inset). The reason for this modification can be deduced from the results in Table 2: for W, most incorrect answers point to N and S. This suggests that these pins are too close to pin W for precise discrimination.  Figure 5(b) shows the prototype developed. Its characteristics are identical to those of prototype 2. This version is also wearable and is meant again for the right foot.

### 5.2. Evaluation

#### 5.2.1. Study Participants and Experimental Procedure

A new group of 20 undergraduate students (15 men and 5 women) participated in the experiment. None of them had tried any of the two previous prototypes. All gave their consent in agreement with the university ethics guidelines. Subjects were healthy sighted with no known impairments in tactile sensory or cognitive functions. Their ages ranged from 20 to 22 years with an average age of 20.8.

The same procedure was followed: subjects were seated wearing socks with the interface on the right foot. There was no previous knowledge of any of the aspects of the experiment. A short familiarization time with the interface was granted prior to the test. Vibrations were at 55 Hz.

#### 5.2.2. Direction Recognition Experiment

The purpose of this test was to determine whether the subjects could obtain even better recognition rates using the third prototype.

*Method.* The same encoding scheme was tested with prototype 3: three consecutive short vibrations in the corresponding contact pin, then a short vibration in the opposite contact pin, and again a short vibration in the correct contact pin (Figure 4).

As with the two previous prototypes, the set of 14 directions was presented in one trial. Subjects were asked to report the direction perceived with no time restriction. Upon request, they could have the direction pattern refreshed on the interface.

*Results*. Subjects required from 3 to 5 min of training to get used to the patterns. Results obtained are presented in confusion matrix in Table 3. The average recognition rates were 100%, 97.78%, 88.89%, and 90% for N, S, W, and E, respectively. The average accuracy of confusion matrix (Table 3) is 94.2%. Note that recognition rates improved for N, S, and W while for E, it was practically the same as in prototype 2. Moving pin S to the tibial area greatly improved user perception.

**Figure 6:**
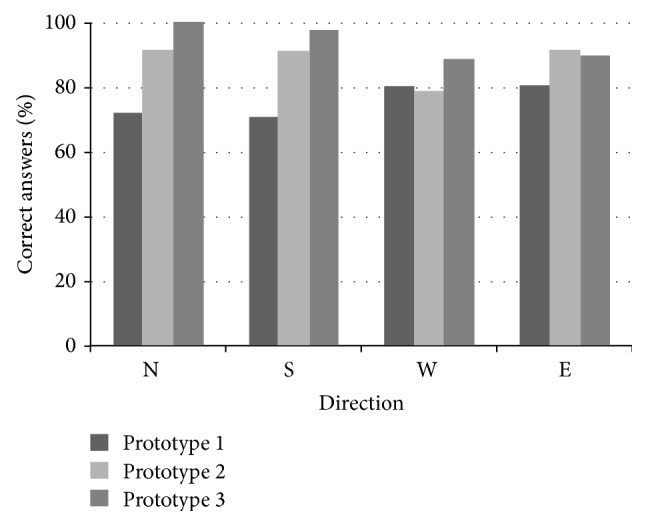
Comparison of performance between prototypes and tactile patterns.

Figure 6 compares the results obtained between prototypes and tactile patterns. Note that both technological and rendering improvements led progressively to better recognition rates.

#### 5.2.3. Navigation in Space

This experiment aims to determine whether the subjects could actually navigate in a structured environment using the directions provided by the third prototype.

*Method*. A camera-based tracking platform was set for this experiment. It consisted of a camera placed 4 m above the ground surface that recorded RGB video. The acquired video was later processed in a PC for subject tracking.

Fifteen (13 men and 2 women) of the 20 subjects of the last group remained available three weeks after the direction recognition test and were invited to participate in this experiment. They had already tried prototype 3 and were familiar with the directional patterns.

The following intuitive protocol was used: North for moving forward, South for moving backward, West for turning left, and East for turning right. A fifth pattern consisting of two consecutive short vibrations, then a pause, and then two consecutive short vibrations (the typical pattern for SMS alerts in mobile phones) was used for indicating to stop. Directions were provided by a computer located outside the navigation environment.

During the test, subjects were blindfolded so that no cue from sight could be obtained. Four different navigational environments (E-1 to E-4) were proposed to the subjects who were totally naive about their structure prior to and during the test.

Subjects were asked to move according to the pattern felt. They had no time restriction to complete the test and, upon request, they could have the directional instruction refreshed on the interface. The navigation time was recorded for each participant.

**Figure 7:**
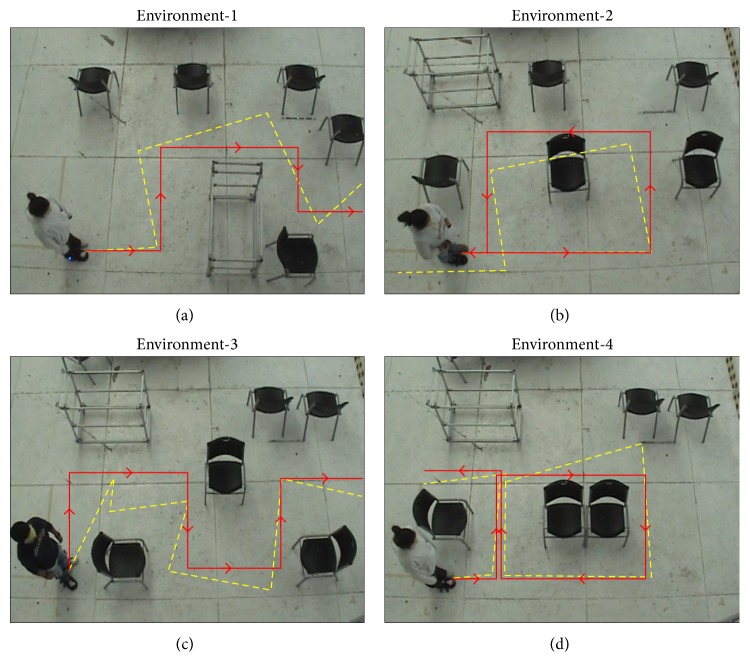
Examples of subject performance in the navigation task. The solid red line represents the ideal trajectory traced by the guiding computer. The broken yellow line is the actual trajectory followed.

*Results*. All subjects were capable of following the navigational instructions and successfully completed the task.  Figure 7 shows a representative example of subject performance per proposed environment. In E-1, E-2, and E-4, subjects had no error following the instructions. In E-3, subject misunderstood E for S and moved backward instead of turning right before being corrected by the computer. Note that, without the visual reference, most sighted subjects fail to walk in straight line: in E-2, despite following the instructions correctly, subject bumped into the obstacles once.

**Figure 8:**
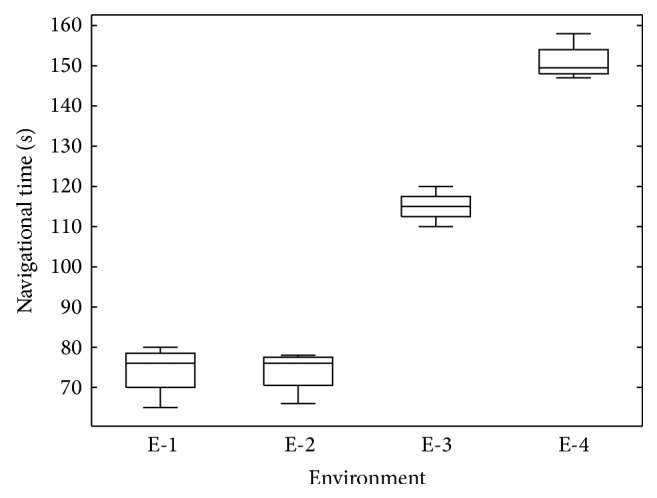
Box plot analysis for the navigational times by environment.

Figure 8 compares the navigational times of the four environments. All four environments were tested by four subjects each (only one subject participated twice navigating E-1 and E-4). Median navigational times were 76, 75, 115, and 149 s for E-1, E-2, E-3, and E-4, respectively. Note that E-1 and E-2 involve the same number of directions and were completed in similar times. The structures of E-3 and E-4 were more complex and required higher navigational times.

Results are undoubtedly encouraging: they suggest that it is feasible to exploit tactile-foot stimulation for directional navigation in space. However, navigational times reported in Figure 8 may raise questions about the practicality of the approach. Note that the proposed environments involve a maze-like structure and require a large number of navigational instructions for completion: 13 instructions were provided to navigate E-1 and E-2, 16 instructions for E-3, and 19 for E-4 (example for E-1:* forward - stop - turn left - forward - stop - turn right - forward - stop - turn right - forward - stop - turn left - forward*). Understanding this amount of information and acting upon it in the reported times seem reasonable. The fact of testing with sighted subjects with no experience in nonvisual travel is also to be considered: motion is performed slower and with certain fear. Outdoor navigation by visually impaired users is expected to be much faster: fewer instructions (only when needed) and natural fearless walking.

## 6. Conclusions

This paper has reported our work and progresses on wearable electronic interfaces for the foot. Using vibrating motors, three simple, low cost, and easy/fast to assemble/maintain designs have been proposed to stimulate the plantar surface and transmit tactile information to the user.

The paper also presented the evolution and technological improvements between designs and prototypes. Lessons learned from the two previous prototypes led to the design of a third optimized version that exhibits significant advances in tactile-foot stimulation.

The pertinence of these advances was evaluated through a perceptual experiment involving direction recognition. Rates show that people understand easier directional information when the medial, lateral, and tibial plantar areas are stimulated and with patterns providing reference points rather than motion.

Real-time navigation in space based on directions was also evaluated. Results show that people are capable of navigating environments using the directions provided by our tactile interfaces for the foot.

The potentials of tactile-foot stimulation and vibrotactile interfaces for the foot can be particularly attractive for assisting people with visual impairment: an unobtrusive, inconspicuous, inexpensive, and fully wearable device that provides intuitive navigational information. Such system would not intend to replace the primary aids but to enhance independent travel by providing directions useful to reach a destination easier and faster. Our future work looks to integrate our third interface with commercially available GPS devices. This will open the possibility of testing in outdoor scenarios.

An important concern on tactile-foot stimulation is cognitive load. Many subjects expressed that vibrating patterns were easy and intuitive but that they needed a certain level of concentration in order to fully and quickly understand them. This could be a limitation for real-life outdoor use: (1) a continuous state of concentration will eventually fatigue users and (2) there are a number of environmental variables that will surely distract the user from the device. Perception and navigational decisions may be affected or slowed down.

**Figure 9:**
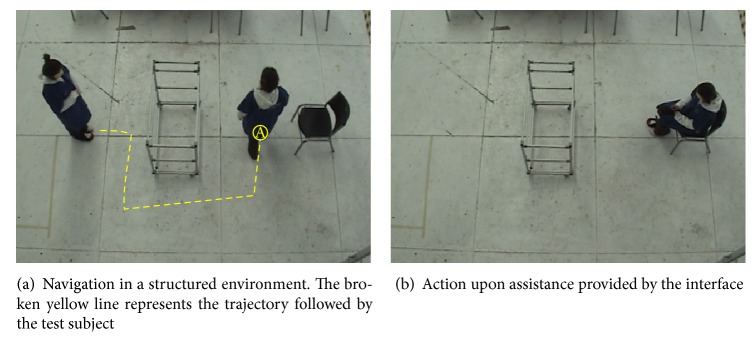
Situational awareness assistance during navigation.

Current work focuses on the design and use of new tactile patterns that represent more complex ideas than just directions. These patterns intend to provide situational awareness assistance during navigation.  Figure 9 illustrates this concept. A blindfolded subject navigates a structured environment as described in Section 5.2.3. At point A, the interface displays obstacle to your left and chair to your right (Figure 9(a)). Subject acts accordingly (Figure 9(b)).

Perceptual studies with voluntary subjects who are visually impaired have been already conducted in the past with our first prototype [9]. Results showed that tactile-foot feedback seems easier to understand for this population. Even better results can be expected from this population with the second and third optimized prototypes presented in this paper.

## Figures and Tables

**Table 1:** Direction recognition rates with prototype 1.

		Answered (%)			
		North	South	West	East
Presented	North	**71.92**	7.02	14.03	7.02
	South	6.57	**71.05**	9.21	13.16
	West	7.9	7.9	**80.24**	3.95
	East	3.51	10.52	5.26	**80.7**

**Table 2:** Direction recognition rates with prototype 2.

		Answered (%)			
		North	South	West	East
Presented	North	**91.65**	1.67	5	1.67
	South	5	**91.25**	1.25	2.5
	West	8.75	8.75	**78.75**	3.75
	East	0	6.67	1.67	**91.65**

**Table 3:** Direction recognition rates with prototype 3.

		Answered (%)			
		North	South	West	East
Presented	North	**100**	0	0	0
	South	2.22	**97.78**	0	0
	West	0	0	**88.89**	11.11
	East	1.67	1.67	6.66	**90**
